# Supplementary material for: Who Thrives in Medical School? Intrinsic Motivation, Resilience, and Satisfaction Among Medical Students
Source: Healthcare (Basel). 2025 Nov 25;13(23):3049. doi: 10.3390/healthcare13233049 (PMC12691725; doi:10.3390/healthcare13233049)
Supplement: Supplementary file 1 [file healthcare-13-03049-s001.zip › healthcare-3957333-supplementary.pdf]

SUPPLEMENTARY MATERIAL 1

Satisfaction with studying medicine questionnaire

Rate your satisfaction with the following areas of studying. If you have not had experience in some of them yet, select the "not applicable" option.

	very dissatisfied	dissatisfied	partly dissatisfied	I have no opinion	partly satisfied	satisfied	very satisfied	not applicable
Overall satisfaction with medical studies								
The mere fact of being admitted to medical studies								
Studying at your current university								
Mode of studies - full-time/part-time								
My knowledge level								
My practical skills								
Extra activity during studies (e.g. scientific, social, artistic, sports)								
The amount of learning material								
Time spent on studies								
Relationships with other medical students								
Peer relationships (outside the medical studies environment)								
Romantic relationships								
Relationships with patients								
Relationships with lecturers								
Relationships with the medical staff (apart from those who hold classes)								
Theoretical classes - lectures, seminars								
Practical classes without patients - practicals, laboratories								
Practical classes with patients								
Student internships								

Validation of the satisfaction with studying medicine questionnaire

As the satisfaction with studying medicine questionnaire was created by the authors, its partial validation was carried out. Due to the fact that this was a post-factum validation, certain stages of typical analysis couldn't be performed.

To evaluate the internal consistency of the questionnaire, the Cronbach-alpha coefficient and the McDonald's-omega reliability coefficient were used. The exploratory factor analysis was performed in order to confirm its unidimensionality. The results of the satisfaction questionnaire in the studied groups are presented in Table 1.

Table S1 The results of the satisfaction with studying medicine questionnaire in the studied groups

**Items**	**Median (IQR)**		
	**1st year students**	**4th year students**	**6th year students**
Overall satisfaction with medical studies	6 (5-6.5)	5 (3-6)	5 (2-6)
The mere fact of being admitted to medical studies	7 (6-7)	6.5 (6-7)	7 (6-7)
Studying at current university	6 (6-7)	6 (5-7)	5 (3-6)
Mode of studies (full-time vs part-time)	6 (6-7)	7 (5.25-7)	6 (5-7)
Level of knowledge	5 (4-6)	5 (3-6)	5 (3-5)
Practical skills	-	3 (2-5)	3 (1-5)
Extra activity during studies (e.g. scientific, social, artistic, sports)	4 (3-6)	5 (3-6)	5 (3-6)
Amount of learning material	3 (2-5)	4 (3-5)	3 (2-5)
Time spent on studies	3 (2-5)	5 (3-5)	3 (2-5)
Relationships with other medical students	6 (4-6)	5 (3-6)	5 (3-6)
Peer relationships (outside the medical studies environment)	6 (5-7)	6 (5-6)	5 (4-6)
Romantic relationships	5 (3-7)	5 (3-7)	6 (3-7)
Relationships with patients	-	6 (5-6)	6 (5-6)
Relationships with lecturers	5 (4-6)	5 (5-6)	5 (4-6)
Relationships with the medical staff	-	5 (4-6)	5 (3-6)
Theoretical classes	5 (4-6)	5 (3-5)	4 (2-5)
Practical classes without patients	6 (5-6)	5 (3-6)	4 (2-5)
Practical classes with patients	-	5 (4-6)	5 (3-6)
Student internships	-	5 (4-6)	5 (3-6)

IQR - interquartile range

The Cronbach-alpha coefficient and the McDonald's-omega reliability coefficient for the satisfaction questionnaire are displayed in Table 2. The values obtained with both methods are similar and suggest high internal consistency of the questionnaire.

Table S2 Reliability analysis results

	**Cronbach-alpha**	**McDonald's-omega**
Satisfaction with studying medicine questionnaire	0.90	0.92

Table 3 shows item-total correlation and how Cronbach-alpha coefficient changes after excluding each item. All correlations are >0,3 and Cronbach-alpha coefficient decreases slightly or remains unchanged after items are removed, which suggests leaving all questions within the questionnaire is justified.

Table S3 Item-total correlation and Cronbach-alpha coefficient changes

Items	Item-total correlation	Cronbach-alpha after excluding item
Overall satisfaction with medical studies	0.77	0.88
The mere fact of being admitted to medical studies	0.45	0.89
Studying at current university	0.68	0.89
Mode of studies (full-time vs part-time)	0.33	0.90
Level of knowledge	0.55	0.89
Practical skills	0.63	0.89
Extra activity during studies (e.g. scientific, social, artistic, sports)	0.38	0.90
Amount of learning material	0.62	0.89
Time spent on studies	0.62	0.89
Relationships with other medical students	0.54	0.89
Peer relationships (outside the medical studies environment)	0.56	0.89
Romantic relationships	0.33	0.90
Relationships with patients	0.59	0.89
Relationships with lecturers	0.62	0.89
Relationships with the medical staff	0.63	0.89
Theoretical classes	0.64	0.89
Practical classes without patients	0.66	0.89
Practical classes with patients	0.70	0.89
Student internships	0.61	0.89

Assessment of unidimensionality of the satisfaction questionnaire is presented in Table 4. As the CFI value indicated the possibility of improving model fit, further factor analysis was performed. In order to confirm whether any particular factors could be detected within the questionnaire, four additional models were created. The Cronbach-alpha coefficients and the McDonald's-omega reliability coefficients for each model and its factors are displayed in Table 5.

Table S4 Assessment of unidimensionality of the satisfaction with studying medicine questionnaire

Unidimen-sionality index	tau	Cronbach-alpha	Correlation mean*	Correlation median**	CFI	ECV
0.77	0.84	0.9	0.32	0.29	0.61	0.72

*mean of correlations between items
**median of correlations between items
CFI - comparative fit index
ECV - explained common variance

Table S5 Reliability analysis for tested models

Model	Factor	Cronbach-alpha	McDonald's-omega
1	1	0.87	0.88
	2	0.83	0.83
	3	0.87	-
2	1	0.83	0.84
	2	0.9	-
	3	0.88	-
	4	0.85	0.87
3	1	0.8	0.82
	2	0.9	-
	3	0.88	-
	4	0.85	0.87
	5	0.66	0.68
4	1	0.87	0.87
	2	0.9	-
	3	0.88	-
	4	0.66	0.68
	5	0.64	0.65
	6	0.79	0.8

The Cronbach-alpha coefficients and the McDonald's-omega reliability coefficients for all models are lower when compared to the original version with all items put together, which suggests that the presented satisfaction questionnaire has no hidden constructs.

SUPPLEMENTARY MATERIAL 2

Motivation to study medicine questionnaire

Rate the extent, to which you agree with the following statements:

Choosing to study medicine was:

	I definitely disagree	I disagree	I rather disagree	I have no opinion	I rather agree	I agree	I definitely agree
My conscious decision							
A decision made based on others' expectations towards me							

I chose to study medicine primarily because of:

	I definitely disagree	I disagree	I rather disagree	I have no opinion	I rather agree	I agree	I definitely agree
A clear and secure future							
My interests							
High income in the future							
Social prestige							
Knowledge useful in private life							
My family members work in healthcare							
I want to feel needed by others							

Are you studying medicine for any other reason not listed above?

…

Validation of the motivation to study medicine questionnaire

As motivation to study medicine questionnaire was created by the research team, its partial validation was carried out. Due to the fact that this was a post-factum validation, certain stages of typical analysis couldn't be performed.

To evaluate the internal consistency of the questionnaire, the Cronbach-alpha coefficient and the McDonald's-omega reliability coefficient were used. The exploratory factor analysis was performed in order to confirm its unidimensionality. The results of the motivation questionnaire in the studied groups are presented in Table 1.

Table S1 The results of the motivation to study medicine questionnaire in the studied groups

Items	Median (IQR)		
	**1st year students**	**4th year students**	**6th year students**
My conscious decision	7 (6-7)	7 (6-7)	7 (5-7)
A decision made based on others' expectations towards me	2 (1-3)	2 (1-3)	2 (1-5)
A clear and secure future	6 (5-7)	6 (5-7)	6 (5-7)
My interests	7 (6-7)	6 (5.25-7)	6 (5-7)
High income in the future	6 (5-7)	5 (5-6)	6 (5-6)
Social prestige	5 (3-6)	5 (3-6)	5 (3-6)
Knowledge useful in private life	6 (4.5-7)	5 (4-6)	6 (5-7)
My family members work in healthcare	1 (1-3)	1 (1-3)	1 (1-5)
I want to feel needed by others	6 (4-7)	6 (5-7)	6 (4-6)

IQR - interquartile range

The Cronbach-alpha coefficient and the McDonald's-omega reliability coefficient for the motivation questionnaire are displayed in Table 2. The values obtained with both methods suggest low internal consistency of the questionnaire in its original version.

Table S2 Reliability analysis results – original version of questionnaire

	**Cronbach-alpha**	**McDonald's-omega**
Motivation to study medicine questionnaire	0.54	0.13

Table 3 shows item-total correlation and how Cronbach-alpha coefficient changes after excluding each item. Presented results suggest that particular items should be excluded in order to improve reliability of the motivation questionnaire.

Table S3 Item-total correlation and Cronbach-alpha coefficient changes – original version of questionnaire

**Items**	**Item-total correlation**	**Cronbach-alpha after excluding item**
My conscious decision	0.21	0.54
A decision made based on others' expectations towards me	- 0.15	0.59
A clear and secure future	0.6	0.52
My interests	0.38	0.53
High income in the future	0.54	0.56
Social prestige	0.6	0.52
Knowledge useful in private life	0.52	0.52
My family members work in healthcare	0.16	0.62
I want to feel needed by others	0.27	0.56

For further analysis, two items were removed. The Cronbach-alpha coefficient and the McDonald's-omega reliability coefficient for the improved version of motivation questionnaire are displayed in Table 4. The values obtained with both methods suggest moderate internal consistency of the questionnaire.

Table S4 Reliability analysis results – improved version of questionnaire

	**Cronbach-alpha**	**McDonald's-omega**
Motivation to study medicine questionnaire	0.62	0.6

Table 5 shows item-total correlation and how Cronbach-alpha coefficient changes after excluding each item in the improved version of questionnaire. All correlations are >0,3, which suggests leaving all questions within the questionnaire is justified.

Table S5 Item-total correlation and Cronbach-alpha coefficient changes – improved version of questionnaire

Items	Item-total correlation	Cronbach-alpha after excluding item
My conscious decision	0.34	0.62
A clear and secure future	0.55	0.56
My interests	0.49	0.59
High income in the future	0.48	0.59
Social prestige	0.55	0.55
Knowledge useful in private life	0.48	0.57
I want to feel needed by others	0.31	0.63

Assessment of unidimensionality of the improved version of motivation questionnaire is displayed in Table 6. As presented results indicated the possibility of improving model fit, further factor analysis was performed. In order to confirm whether any particular factors could be detected within the questionnaire, two additional models were created. The Cronbach-alpha coefficients and the McDonald's-omega reliability coefficients for each model and its factors are displayed in Table 7.

Table S6 Assessment of unidimensionality of the motivation to study medicine questionnaire

Unidimen-sionality index	tau	Cronbach-alpha	Correlation mean*	Correlation median**	CFI	ECV
0.36	0.57	0.62	0.2	0.17	0.55	0.45

*mean of correlations between items
**median of correlations between items
CFI - comparative fit index
ECV - explained common variance

Table S7 Reliability analysis for tested models

Model	Factor	Cronbach-alpha	McDonald's-omega
1	1	0.69	0.71
	2	0.54	0.55
2	1	0.67	-
	2	0.67	-
	3	0.45	0.46

The model with two factors proved to have the highest reliability coefficients when compared to one factor- and three factors models. Table 8 displays its further analysis.

Table S8 Assessment of unidimensionality of each factor in two factors model of motivation questionnaire

Factor	Unidimen-sionality index	tau	Cronbach-alpha	Correlation mean*	Correlation median**	CFI	ECV
1	0.96	0.96	0.69	0.48	0.52	1	0.96
2	0.78	0.8	0.54	0.26	0.2	0.98	0.82

*mean of correlations between items
**median of correlations between items
CFI - comparative fit index
ECV - explained common variance

Analysis performed on the improved version of motivation to study medicine questionnaire suggests it contains hidden constructs. The exploratory factor analysis showed a possibility to extract two factors, which may refer to external and intrinsic motivation (Table 9).

Table S9 Model with two factors – item details

Factor	Item
1 – external motivation	A clear and secure future
	High income in the future
	Social prestige
2 – intrinsic motivation	My conscious decision
	My interests
	Knowledge useful in private life
	I want to feel needed by others

SUPPLEMENTARY MATERIAL 3

Evaluation of personal circumstances questionnaire

Rate the following aspects of your personal situation:

	Very bad	Bad	Rather bad	I have no opinion	Rather good	Good	Very good
Your financial situation							
Your health							
Life satisfaction in general							
Current levels of stress related to your studies							
Stress related to your studies in the previous years (years 4 and 6)							

SUPPLEMENTARY MATERIAL 4

Self-reported COVID-19 pandemic impact on students' academic performance questionnaire
How have learning conditions during the COVID-19 pandemic affected your academic performance ?

The COVID-19 pandemic did not affect my studies and competences
I have gaps in my knowledge
I lack interpersonal skills
I lack practical skills
I lack self-confidence as a future doctor
I think that my future patients will not trust me
During the pandemic I was still studying in high school
Other

SUPPLEMENTARY MATERIAL 5

Demographic characteristics of participants

**Characteristics**		***n* (%)**
Gender		
	Male	71 (21%)
	Female	263 (79%)
Age		$M = 22.26$, $SD = 2.63$
Place of origin		
	Countryside	72 (22%)
	City up to 100 000 inhabitants	109 (33%)
	City up to 100 000 – 200 000 inhabitants	38 (11%)
	City up to 200 000 – 500 000 inhabitants	40 (12%)
	City over 500 000 inhabitants	75 (22%)
Marital status		
	Not in a formal relationship	147 (44%)
	In an informal monogamous relationship	171 (51.2%)
	In an informal polyamorous relationship	5 (1.5%)
	Married	6 (1.8%)
	Prefer not to say	5 (1.5%)
Number of friends		
	Studying medicine	$M = 3$, $SD = 3$
	Met outside the medical studies environment	$M = 4$, $SD = 3$
Mode of studies		
	Full-time	284 (85%)
	Part-time	50 (15%)
Year of studies		
	1st year	119 (36%)
	4th year	118 (35%)
	6th year	97 (29%)
University		
	Medical University of Gdansk	117 (35%)
	Poznan University of Medical Sciences	87 (26%)
	Medical University of Lodz	33 (9.9%)
	Medical University of Warsaw	19 (5.7%)
	Medical University of Silesia	13 (3.9%)
	Medical University of Lublin	9 (2.7%)
	Medical University of Wroclaw	8 (2.4%)
	Ludwik Rydygier Collegium Medicum in Bydgoszcz (Nicolaus Copernicus University in Toruń)	7 (2.1%)
	Jagiellonian University	6 (1.8%)
	Casimir Pulaski Radom University	6 (1.8%)
	Medical University of Bialystok	5 (1.5%)
	College of Medical Sciences (University of Rzeszow)	5 (1.5%)
	Pomeranian Medical University	5 (1.5%)
	University of Opole	3 (0.9%)
	Andrzej Frycz Modrzewski Krakow University	2 (0.6%)
	University of Warmia and Mazury	2 (0.6%)
	Cardinal Stefan Wyszynski University in Warsaw	2 (0.6%)
	School of Medicine (Jan Kochanowski University)	1 (0.3%)
	Lazarski University	1 (0.3%)
	Maria Sklodowska-Curie Medical University in Warsaw	1 (0.3%)
	University of Zielona Gora	1 (0.3%)
	Mazovian University in Plock	1 (0.3%)
